# Bone tissue engineering using 3D silk scaffolds and human dental pulp stromal cells epigenetic reprogrammed with the selective histone deacetylase inhibitor MI192

**DOI:** 10.1007/s00441-022-03613-0

**Published:** 2022-04-01

**Authors:** Kenny Man, Habib Joukhdar, Xue D. Manz, Mathieu Y. Brunet, Lin-Hua Jiang, Jelena Rnjak-Kovacina, Xuebin B. Yang

**Affiliations:** 1 Biomaterials & Tissue Engineering Group, School of Dentistry, University of Leeds, WTBB, St. James’s University Hospital, Leeds, LS97TF UK; 2 School of Chemical Engineering, University of Birmingham, Birmingham, UK; 3 Graduate School of Biomedical Engineering, University of New South Wales, Sydney, Australia; 4 Department of Pulmonary Medicine, Amsterdam UMC, VU University Medical Centre, Amsterdam, The Netherlands; 5 School of Biomedical Sciences, University of Leeds, Leeds, UK

**Keywords:** Histone deacetylase inhibitor, Epigenetics, Silk scaffolds, Bone tissue engineering, Human dental pulp stromal cells

## Abstract

Epigenetics plays a critical role in regulating mesenchymal stem cells’ (MSCs) fate for tissue repair and regeneration. There is increasing evidence that the inhibition of histone deacetylase (HDAC) isoform 3 can enhance MSC osteogenesis. This study investigated the potential of using a selective HDAC2 and 3 inhibitor, MI192, to promote human dental pulp stromal cells (hDPSCs) bone-like tissue formation in vitro and in vivo within porous *Bombyx Mori* silk scaffolds. Both 2 and 5 wt% silk scaffolds were fabricated and characterised. The 5 wt% scaffolds possess thicker internal lamellae, reduced scaffold swelling and degradation rates, whilst increased compressive modulus in comparison to the 2 wt% silk scaffold. MI192 pre-treatment of hDPSCs on 5 wt% silk scaffold significantly enhanced hDPSCs alkaline phosphatase activity (ALP). The expression of osteoblast-related genes (RUNX2, ALP, Col1a, OCN) was significantly upregulated in the MI192 pre-treated cells. Histological analysis confirmed that the MI192 pre-treated hDPSCs-silk scaffold constructs promoted bone extracellular matrix (ALP, Col1a, OCN) deposition and mineralisation compared to the untreated group. Following 6 weeks of subcutaneous implantation in nude mice, the MI192 pre-treated hDPSCs-silk scaffold constructs enhanced the vascularisation and extracellular matrix mineralisation compared to untreated control. In conclusion, these findings demonstrate the potential of using epigenetic reprogramming and silk scaffolds to promote hDPSCs bone formation efficacy, which provides evidence for clinical translation of this technology for bone augmentation.

**Graphical abstract:**

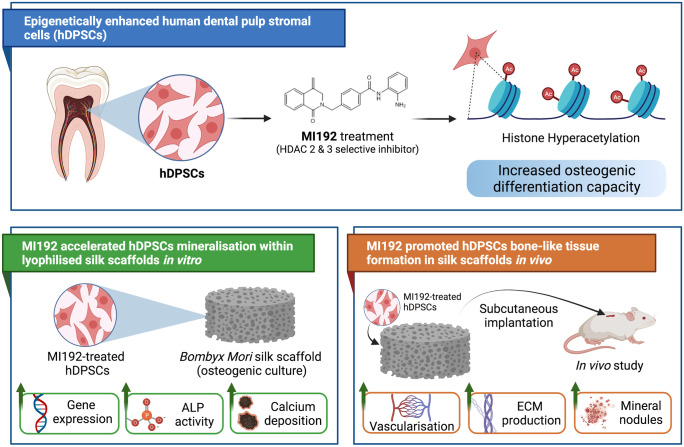

**Supplementary Information:**

The online version contains supplementary material available at 10.1007/s00441-022-03613-0.

## Introduction

The increasing ageing population and traumatic injury creates an enormous healthcare and socioeconomic burden, resulting in a major clinical need for bone tissue (Baroli 2009; Dimitriou et al. 2011; Lawlor and Yang 2019). To date, treating musculoskeletal disorders in the UK costs the National Health Service approximately £5 billion annually (Chance-Larsen et al. 2019). Current gold standard therapies to repair critical-sized bone defects, autografts suffer from limited supply and morbidity at the donor site (Berthiaume et al. 2011; Dimitriou et al. 2011). Due to these limitations, tissue engineering approaches have been explored as an alternative strategy to meet the rising demand for clinically relevant bone tissue. For bone tissue engineering, it is essential to effectively control the lineage-specific differentiation of mesenchymal stromal cells (MSCs) (Amini et al. 2012), and recent technologies such as gene therapy have garnered excitement within the field. However, these methods are associated with safety concerns such as tumourigenesis (Genetics Home Reference 2021; Maroni et al. 2012). Hence, there is a critical calling to develop novel approaches to effectively control MSCs fate for bone augmentation strategies.

Numerous studies have shown that modifying the epigenome impacts cell fate (Hu et al. 2013; Marks 2010; Maroni et al. 2012). Post-translation modifications such as acetylation have been reported to alter the cells’ transcriptional activity by controlling the conformational structure of the chromatin (Tollervey and Lunyak 2012). The acetylation process is tightly regulated by two enzymes, histone acetyltransferase (HAT) and histone deacetylase (HDAC), which mediate the addition or removal of acetyl groups from lysine residues on histone proteins, respectively (Lawlor and Yang 2019). Hyperacetylation of the chromatin leads to a more open chromatin structure exhibiting increased transcriptional activity, whilst deacetylation activity results in chromatin condensation and transcriptional repression (Yang and Seto 2007). Researchers have investigated the possibility of harnessing the transcriptional plasticity of the chromatin via the use of HDAC inhibitor (HDACi) compounds, resulting in hyperacetylation (Huynh et al. 2016; Xu et al. 2007). Several studies have demonstrated the utility of modifying the epigenome with HDACis to enhance the osteogenic capacity of cells (de Boer et al. 2006; Man 2021a; Paino et al. 2014). Although promising results have been observed, the majority of studies have utilised non-selective panHDACis, which can bind to an extensive variety of HDAC isoforms, resulting in reduced differentiation efficacy and unwanted side effects (Yang et al. 2017); consequently, there is an increasing interest in using isoform-specific HDACis (Balasubramanian et al. 2009; Gryder et al. 2012). Studies have indicated that the HDAC3 isoform is causatively linked to osteogenesis due to its repression of the osteogenic transcription factor, Runx2 (Schroeder et al. 2004; Schroeder and Westendorf 2005). It has been reported that the HDAC2 and 3 selective inhibitor — MI192 is capable of enhancing the osteogenic capacity of adipose-derived MSCs (ADSCs) when compared to the use of a panHDACi Trichostatin A (TSA) (Lawlor 2016).

Moreover, the efficacy of MI192 in stimulating osteogenic differentiation and mineralisation of human dental pulp stromal cells (hDPSCs) and human bone marrow stromal cells (hBMSCs) has also been reported (Man et al. 2021b, c). Although the potential of HDACis for bone regeneration has been demonstrated, the majority of studies have evaluated their efficacy in 2D in vitro cultures, which does not replicate the conditions in situ (Abbott 2003; Baker and Chen 2012). Hence, there is a tremendous need to investigate the therapeutic efficacy of these epigenetic modifying compounds in a more physiologically relevant microenvironment.

One of the key challenges for engineering bone tissue is using an appropriate biomaterial to provide suitable mechanical and biological properties that can mimic natural bone matrix and direct de novo tissue formation. Silk scaffolds have been utilised for multiple tissue engineering applications such as cardiac (Patra et al. 2012), neural (Allmeling et al. 2008), cartilage (Saha et al. 2013) and bone tissue engineering (Correia et al. 2012), where it has shown increasing promise. Silk is a natural biomaterial found abundantly and can be easily acquired from silkworms and spiders (Barroso et al. 2021; von Byern et al. 2019; Yoshioka et al. 2019). As well as being naturally abundant, silk possesses numerous advantageous characteristics such as being cytocompatible, easily fabricated, highly porous and biodegradability (Melke et al. 2016; Saha et al. 2013). Due to the plethora of benefits silk provides as a biomaterial, it has been utilised for numerous bone tissue engineering studies. For example, Saha et al. incorporated silk scaffolds with the osteoinductive growth factor bone morphogenetic protein 2 (Saha et al. 2013), whilst Zhao et al. applied an apatite coating to promote the osteoinductive potency of these scaffolds (Zhao et al. 2009). Meinel et al. demonstrated that silk scaffolds could repair critical-sized bone defects within mouse models (Meinel et al. 2005). As silk scaffolds have proven their potential for numerous tissue engineering applications, including bone (Correia et al. 2012; Sun et al. 2021), this biomaterial could provide a suitable platform to facilitate the delivery of MI192 pre-treated MSCs in vivo, supporting de novo bone formation.

**Fig. 1:**
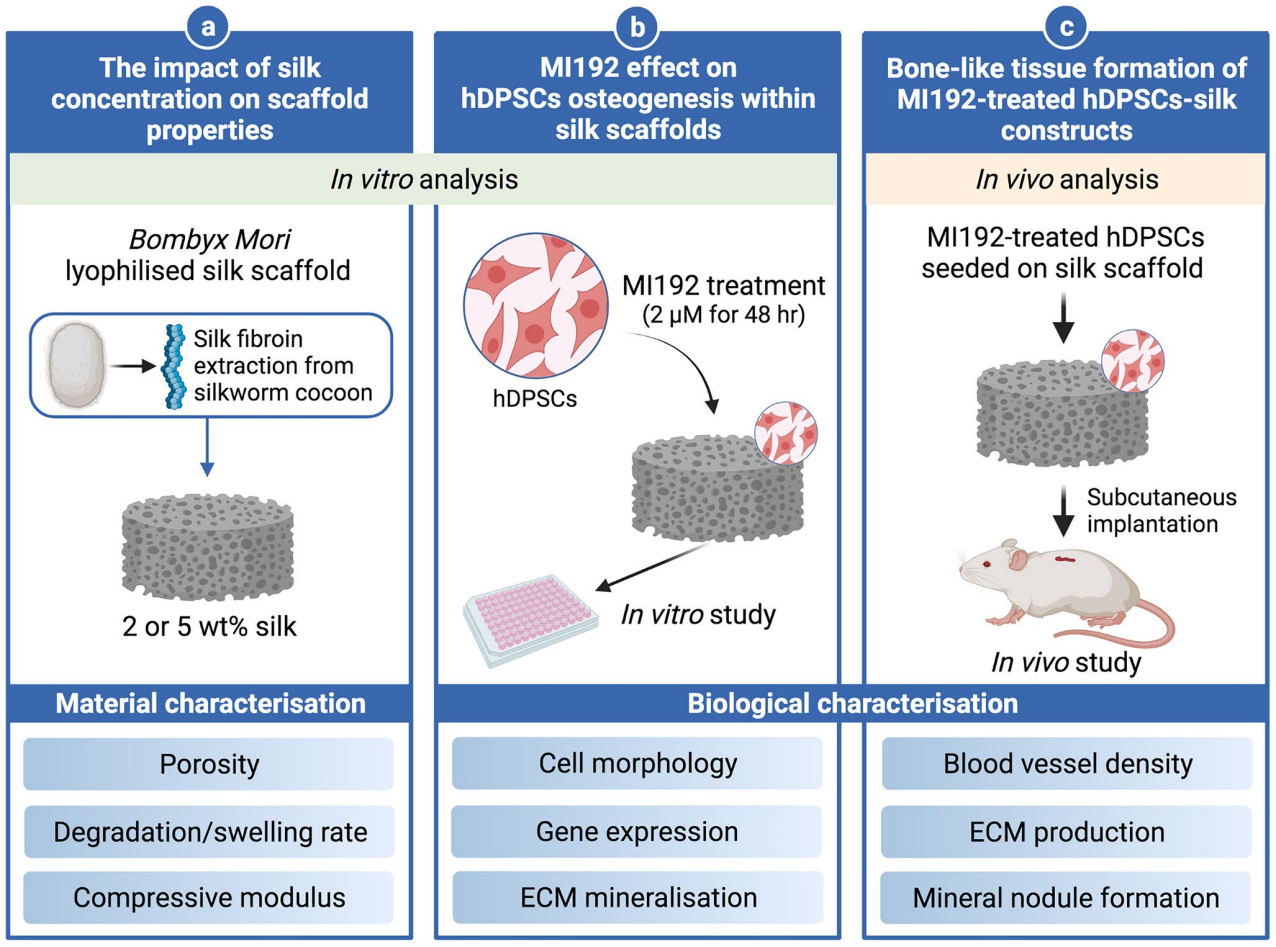
Experimental outline investigating the influence of MI192 pre-treatment on osteogenic differentiation of hDPSCs within 3D silk scaffold in vitro and in vivo. **a** The influence of altering silk concentration on the scaffolds physical properties and osteogenic capacity was evaluated. **b** The effect of MI192 in stimulating hDPSCs mineralisation within silk scaffolds in vitro was investigated. **c** Bone-like tissue formation induced by MI192 pre-treatment of hDPSCs was evaluated following 6 weeks of subcutaneous implantation in mice. (The figure was created with Biorender.com)

Thus, this study aimed to explore the potential of combining MI192 pre-treated hDPSCs with porous *Bombyx Mori*-lyophilised silk scaffolds to enhance bone-like tissue formation in vitro and in vivo (Fig. 1)*.*

## Materials and methods

### Silk fibroin extraction

*Bombyx mori* silk cocoons were purchased from Tajima Shoji Co Ltd (Japan). The silk fibroin solution was prepared (Rockwood et al. 2011) by cutting 5 g of *Bombyx mori* silk cocoons into small pieces, which was boiled in 2 L of 0.02-M sodium carbonate solution for 30 min to remove sericin. Silk fibroin fibres were washed in water and dissolved in 9.3 M lithium bromide (25% wt/v) for 4 h at 60 °C. Silk fibroin solution was dialyzed against water using SnakeSkin™ dialysis tubing (3500MWCO, Thermo Fisher Scientific) for 3 days to remove lithium bromide and centrifuged twice at 8700 RPM to remove debris. Silk fibroin concentration was determined by drying a known volume of the solution and weighing the remaining film. Silk fibroin solution was stored at 4 °C. Silk fibroin is referred to as ‘silk’ in this article.

### Silk scaffold fabrication

According to our previous research, porous silk scaffolds were fabricated by freezing a 2% or a 5% wt/v silk solution (in water) in 24-well tissue culture plates overnight at −20 °C, followed by lyophilisation and autoclaving at 121 °C to induce β-sheet formation for the formation of 2 and 5 wt% porous silk scaffolds (Rnjak-Kovacina et al. 2015). Scaffolds were hydrated in PBS and trimmed to size prior to use.

### Characterisation of silk scaffolds

Dried samples were gold sputter-coated (40 mA, 60 s) and imaged with a field-emission scanning electron microscopy (SEM, Supra55VP, Zeiss, Germany) using a 5 kV electron beam. The compressive mechanical properties of the silk scaffolds were evaluated using an Instron 5543 mechanical tester with a 50 N load cell. Silk scaffolds (Ø8 mm × 4 mm) were compressed to 90% of their original height at a rate of 10 mm/min. Compressive modulus was determined between 10 and 30% strain. Silk scaffold swelling was assessed by incubating samples (Ø8 mm × 4 mm) in water or PBS and quantifying the solution uptake by mass relative to dry scaffolds. In vitro degradation was determined by incubating silk scaffolds (semicircle, Ø15 mm × 5 mm) in 2 U/ml Protease XIV solution at 37 °C for 8 days. Scaffolds were washed in water, dried and weighed at each time point before fresh enzyme solution was added (Rnjak-Kovacina et al. 2015).

### Cell isolation and in vitro expansion

HDPSCs were isolated from extracted impacted third molars obtained with patient consent through the Leeds Dental Institute/School of Dentistry, Skeletal Research Tissue Bank (REC 07/H1306/93) with ethical approval (180,615/KM/173) (El-Gendy et al. 2013). Cells were subsequently cultured in basal medium containing alpha modified minimum essential medium (α-MEM, Lonza, UK) in supplement with 10% foetal calf serum (FCS, Lonza, UK), 2-mM L-glutamine and a mix of 100 units/mL penicillin with 100 μg/ml streptomycin at 37 °C in 5% CO_2_ in the air. Cells were passaged when approaching 80% confluency, and passage 4 was used for this study.

### Cell seeding and osteogenic differentiation on silk scaffolds

Lyophilised silk scaffolds were cut to size (Ø5 × 2 mm) and washed with PBS. Scaffolds were then incubated in α-MEM containing 10% FCS overnight at 37 °C. Prior to adding cells, the scaffolds were washed with plain medium, and excess media within scaffolds was removed and allowed to air dry. HDPSCs were pre-treated with MI192 (2 μM for 48 h) as previously described (Man et al. 2021b). Untreated and MI192 pre-treated cells were seeded statically onto prepared scaffolds (2 × 10^5^ cells per scaffold). Briefly, a cell suspension containing 2 × 10^5^ cells was collected by centrifugation and re-suspended in 20 µl of osteogenic medium (basal medium supplemented with 50-μM L-ascorbic acid 2-phosphate sesquimagnesium salt hydrate (Sigma-Aldrich, UK), 10-mM β-glycerol phosphate (Sigma-Aldrich, UK) and 100-nM dexamethasone (Sigma-Aldrich, UK). A cell suspension (10 µl) was placed on the bottom of a non-adherent 48 well plate (Corning, UK), and the scaffold was placed on top of the cell suspension for 10 min. After this period, the remaining cell suspension (10 µl) was transferred to the top of the scaffold, which was left to adhere for 1 h, prior to the addition of osteoinductive medium into the well. The media was changed every 3–4 days.

Cell viability and morphology on silk scaffolds 24-h post-seeding, and following 6 weeks osteoinduction was evaluated via fluorescent imaging. Briefly, media were replaced with a fresh basal medium containing CellTracker™ Green CMFDA dye and ethidium homodimer-1 (Thermo Scientific, UK) and incubated in the dark for 30 min. After incubation, scaffolds were washed in a fresh basal medium prior to imaging using a confocal laser scanning microscope (Leica, UK).

### Determination of osteogenic gene expression using RT-qPCR

Cell-laden silk constructs were sliced using a sterile scalpel and placed into 500-µl RLT buffer (Qiagen, UK). Samples were vortexed and sonicated for 15 min, which was repeated four times. The lysate solution was transferred into the QIAshredder (Qiagen, UK) and centrifuged in a bench-top centrifuge at 10,000 RPM for 3 min. Cells were lysed following the RNase mini kit (Qiagen, UK) protocol. The concentration/quality of RNA was assessed using a Nanodrop spectrophotometer (Thermo Scientific, UK). RNA was converted to cDNA using a high-capacity RNA to cDNA Kit (Applied Biosystems, UK). The gene expression levels were assessed by RT-qPCR using the TaqMan gene expression assay as previously described (Man et al. 2021b). The cDNA was amplified using TaqMan primers (Supplementary Table 1) in a 20-µl reaction in a 96-well PCR plate (Starlab, UK). Amplification occurred, and the cycle threshold (C_t_) was obtained within a LightCycler 480 qPCR system (Roche, UK). The comparative C_t_ (2^−∆∆Ct^) was used for quantifying the gene expression levels (normalised by the housekeeping gene).

### Alkaline phosphatase specific activity (ALPSA)

Cell-laden silk constructs were washed three times in PBS, then cut into slices using a sterile scalpel. Silk pieces were then transferred into 500 µl 0.1% Triton X-100 in PBS and were vortexed/sonicated for 5 min and then frozen at − 80 °C. Samples were then thawed at 37 °C, and the lysing/freeze/thaw process was repeated four times. Following this, samples were centrifuged at 10,000 × *g* for 10 min at 4 °C, and the lysate was collected and utilised to determine ALPSA. ALP quantification was determined using the 4-nitrophenyl phosphate liquid substrate system (pNPP, Sigma-Aldrich, UK). DNA content was assessed using PicoGreen (Life Technologies, UK) as per the manufacturer’s protocol. ALPSA was calculated by dividing the total ALP quantified by the total DNA content of that sample determined by PicoGreen DNA assay (nmol ALP/h/μg of DNA).

### Subcutaneous implantation of cell-laden silk constructs

All procedures used were conducted under the approval of the University of Leeds Ethics Committee and under the UK Home Office project license (PPL: 70/8549). All animals in this study were 6-week-old CD1 nude male mice. A total of 5 wt% silk scaffolds were seeded with MI192 pre-treated and untreated hDPSCs as previously described. Following 24-h post-seeding, the constructs were implanted in subcutaneous pockets of each mouse under general anaesthesia using isoflurane. At 6 weeks post-surgery, animals were euthanized, and the constructs were retrieved and fixed in 10% neutral buffered formalin (NBF, Cellpath, UK). Samples were processed for histology and immunohistochemical analysis.

### Histological analysis

Fixed samples were embedded in paraffin and sectioned (4 μm) using a microtome (Leica, UK). Haematoxylin and eosin (H&E) and picrosirius red/alcian blue staining were undertaken to visualise cell distribution and collagen/glycosaminoglycans (GAGs) deposition, respectively. Calcium deposition and mineralisation were assessed via alizarin red (Millipore, UK) and Von Kossa staining (Atom Scientific, UK). Stained samples were captured under an Olympus BX50 microscope and analysed using NIS Elements BR software (Ver. 3.0). Protein deposition was assessed using an EnVision™ Detection Systems Peroxidase/DAB, Rabbit/Mouse kit (Dako, UK). Briefly, sections were placed in a PBS bath before incubation with ‘Dual Endogenous Enzyme Block’ from the EnVision™ kit for 10 min. Sections were washed in PBS bath for 5 min and then blocked with 20% normal goat serum (Dako, UK) in PBS for 30 min. Primary antibodies (Supplementary Table 2) were added to the samples at the desired concentration in 1% BSA (Sigma-Aldrich, UK) in PBS and left to incubate overnight at 4 °C. Sections were washed in PBS for 10 min before incubation with the secondary HRP-conjugated goat anti-rabbit IgG antibody to the samples for 30 min. After washing again in PBS for 5 min, the Dako DAB developing solution was added and incubated for 10 min prior being immersed in Harris Haematoxylin for 20 s. Samples were then examined under an Olympus BX50 microscope.

In vivo degradation was calculated from 16 H&E stained images taken from the centre of each scaffold. Degradation was semi-quantified by tracing the perimeter of internal lamellae present in the scaffold using ImageJ (National Institutes of Health, USA) (Lin et al. 2020). Similarly, mean blood vessel number was measured via ImageJ (identified through a clear lumen containing red blood cells) (16 images per sample per group).

### Statistical analysis

Data expressed as mean ± standard deviation (SD). Quantitative data were statistically analysed by ANOVA multiple comparisons test with Tukey modification using SPSS software (IBM Analytics, version 21). NS: *P* > 0.05; *P ≤ 0.05; **P ≤ 0.01 and ***P ≤ 0.001.

## Results

### Porosity and pore morphology of lyophilised silk scaffolds

**Fig. 2:**
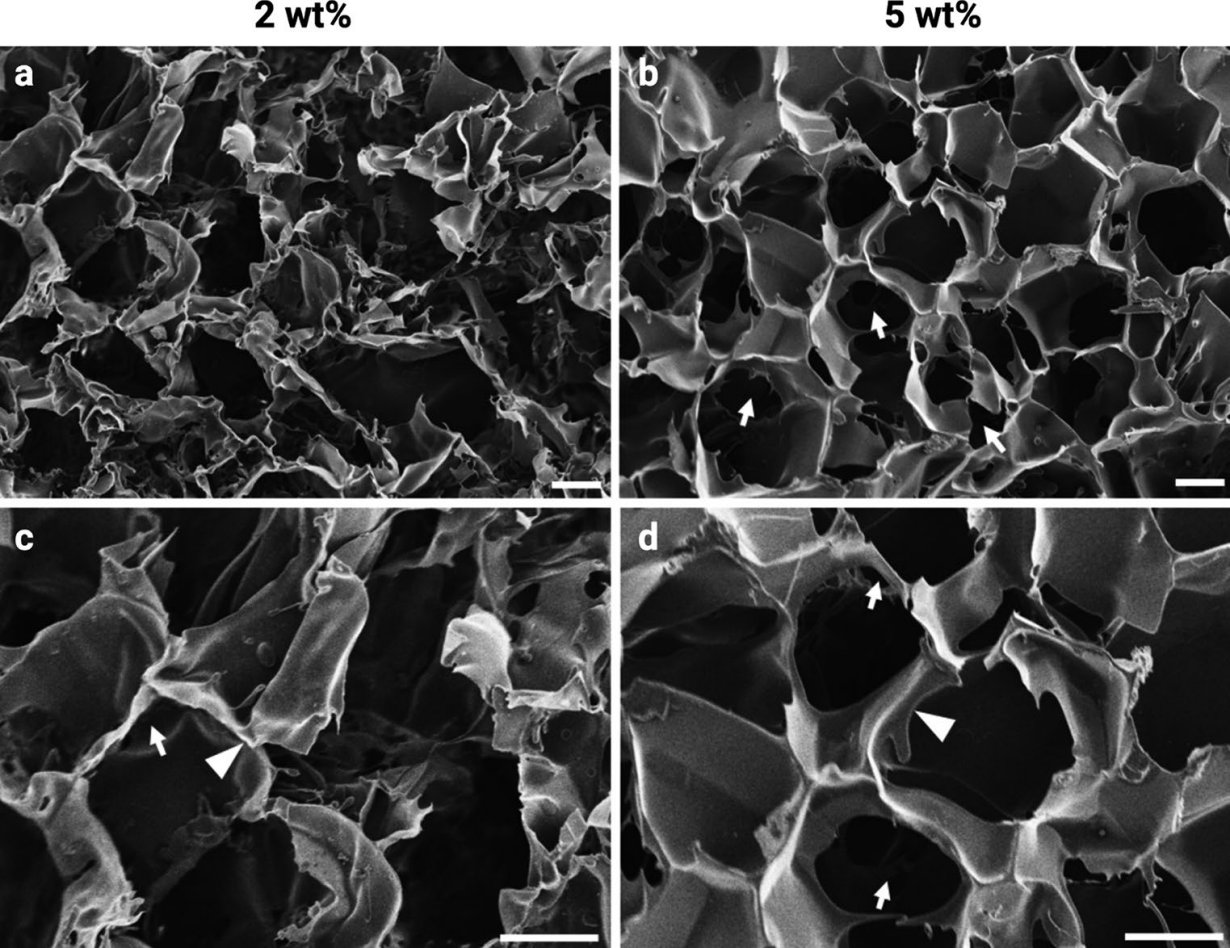
SEM micrographs showing the porosity, pore morphology and lamellae network in 2 wt% (**a**, **c**) and 5 wt% (**b**, **d**) silk scaffolds. The white arrows indicate the interconnection between the pores. The white arrowheads indicate the thin sheet-like lamellae between the pores. Scale bar, 100 μm

SEM analysis of the 2 wt% (Fig. 2a, c) and 5 wt% (Fig. 2b, d) silk scaffolds was conducted to evaluate the porosity, pore morphology and lamellae network. The micrographs revealed a highly porous structure in both wt% groups, with a network of thin interconnected sheet-like lamellae (white arrow). The thickness of the lamellae sheets tends to increase with greater silk concentration in the scaffold.

### Effects of silk concentrations on scaffold swelling, degradation and mechanical properties

**Fig. 3:**
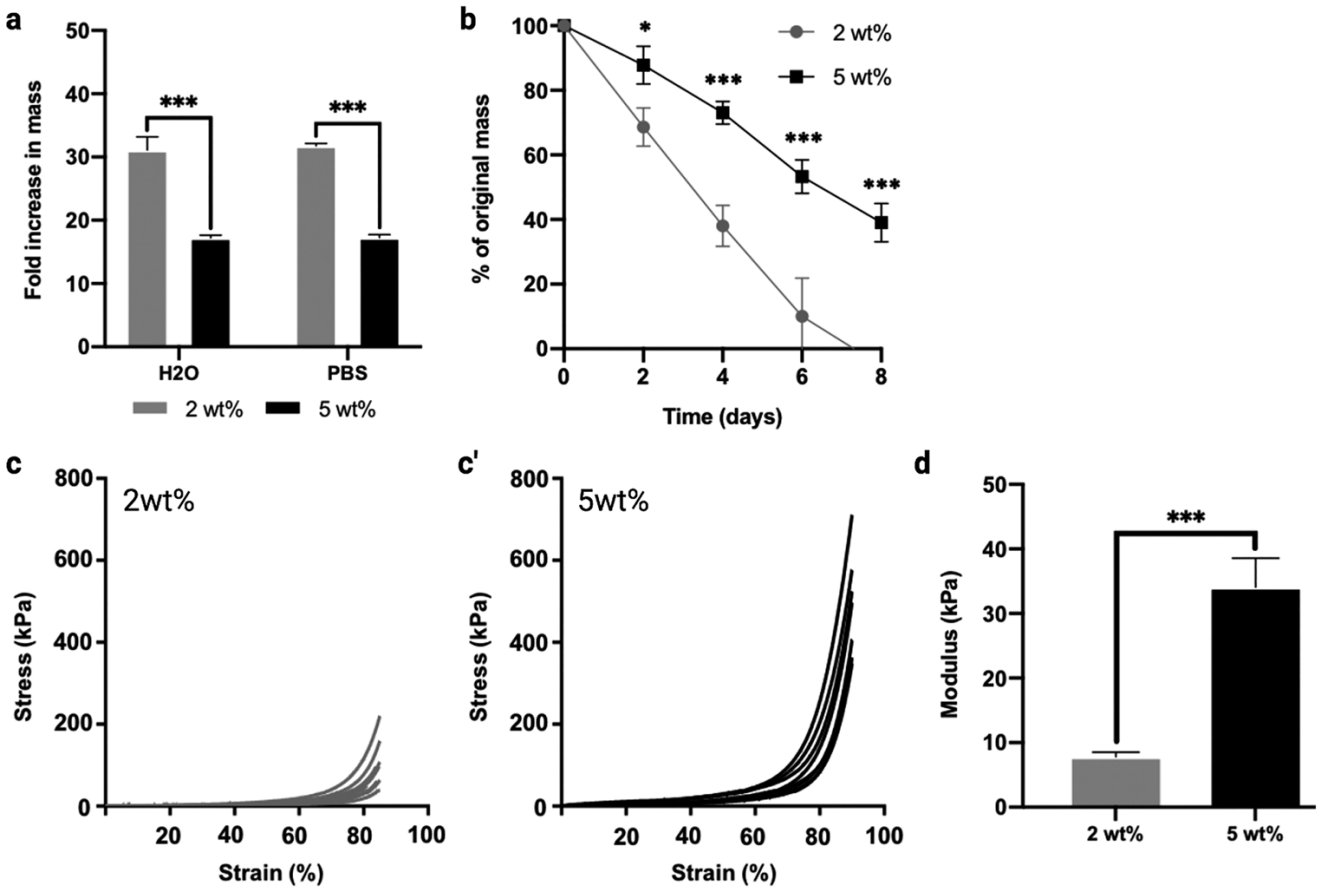
Effects of silk concentration on swelling capacity (**a**), in vitro degradation (**b**) and compressive modulus (**c**, **c’**, **d**). Data expressed as mean ± SD (*n* = 3). *P ≤ 0.05 and ***P ≤ 0.001

Scaffold swelling in water and PBS showed a marked increase in mass following hydration of dry scaffolds (Fig. 3a). A total of 5 wt% scaffolds exhibited a significant decrease (~ 1.9 times) in the swelling capacity when compared to the 2 wt% scaffolds (*P* ≤ 0.001). A total of 2 wt% silk scaffolds absorbed 32.5 ± 2.0 and 31.5 ± 1.1 times their mass in water and PBS, respectively. A total of 5 wt% scaffolds absorbed 16.9 ± 0.5 and 17.2 ± 0.4 times their mass in water and PBS, respectively.

To evaluate the effects of silk concentration on lyophilised scaffold degradation, the in vitro degradation of the scaffolds was investigated at days 2, 4, 6 or 8 after exposure to Protease XIV (Fig. 3b), showing the percent remaining mass. At each time point, the remaining mass of 2 wt% silk scaffolds was significantly lower than that of 5 wt% scaffolds (*P* ≤ 0.05–0.001 respectively), demonstrating that the degradation of 2 wt% silk scaffolds was significantly faster in vitro relative to that of 5 wt% silk scaffolds.

Figure 3c, c’ and d show the compressive modulus of the silk scaffolds. When compressed to 90% of their original size, 5 wt% silk scaffolds were stronger and less elastic than that of 2 wt% silk scaffolds. The compression modulus of 5 wt% scaffolds was 34.06 ± 4.52 kPa and was significantly higher (4.35 times) than that of 2 wt% silk scaffolds at 7.82 ± 0.70 kPa (*P* ≤ 0.001).

### Effects of silk scaffold concentration on MI192 pre-treated hDPSCs viability and osteogenic differentiation in vitro

**Fig. 4:**
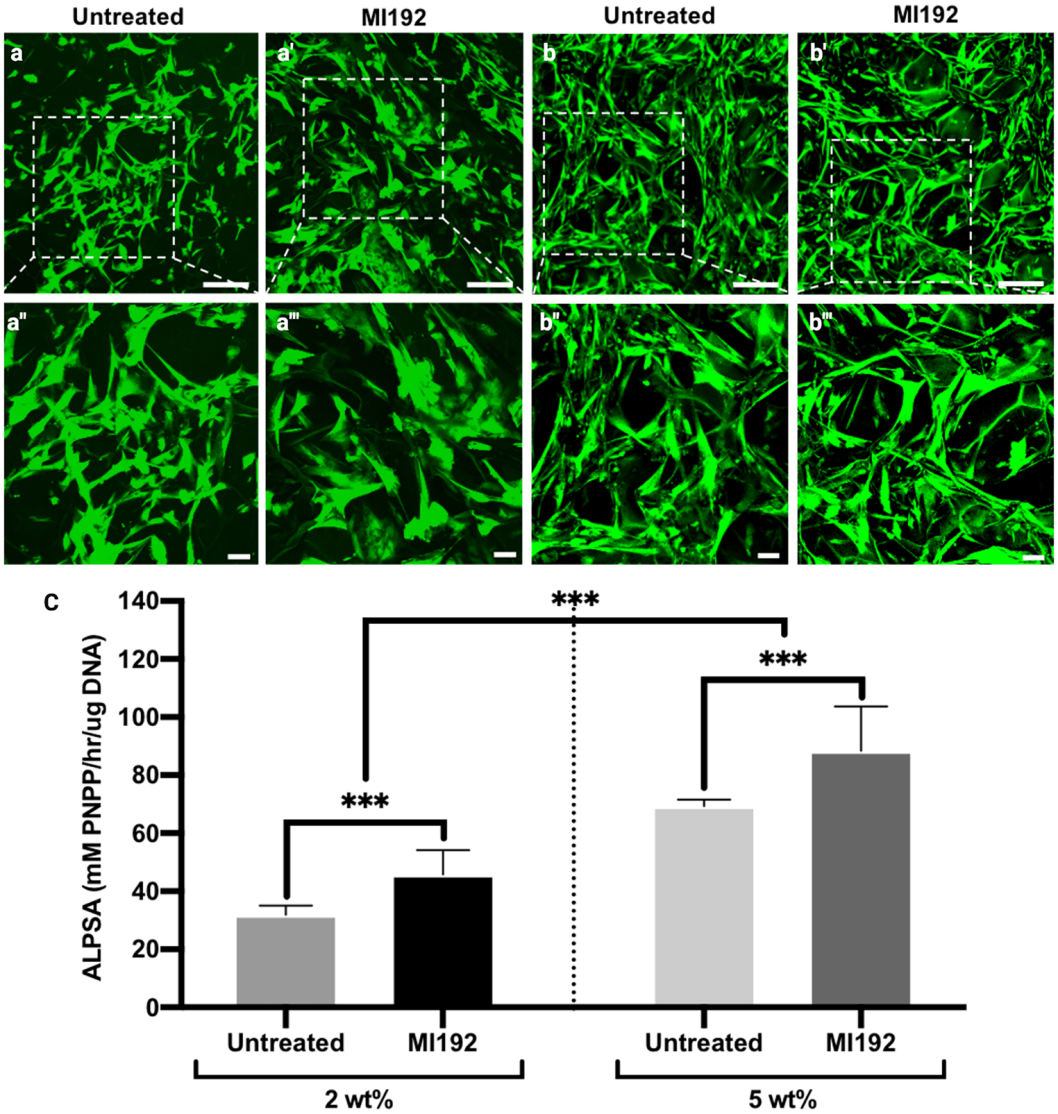
The influence of silk concentrations on hDPSC viability and osteogenic capacity. Live fluorescent staining of untreated/MI192 pre-treated hDPSCs after 24 h (**a**–**a’’’**) and 6 weeks (**b**–**b’’’**) in osteogenic culture. Low magnification (the top panel) scale bars = 200 μm, and high magnification (the second panel) scale bars = 50 μm. **c** ALPSA of MI192 pre-treated cells on silk scaffolds following 2 weeks in osteogenic culture. Data represented as mean ± SD (*n* = 3). ***P ≤ 0.001

Silk scaffolds were seeded with MI192 pre-treated or untreated hDPSCs and labelled with CFMDA to visualise the cells within the scaffolds (Fig. 4a–a’’’). The interconnectivity of the scaffolds was further confirmed by fluorescent staining, where cells were observed attached to the internal lamellae network of the scaffolds. After 24-h post-seeding, viable cells were homogeneously distributed throughout the scaffolds, with the majority of the cells possessing an elongated morphology. The MI192 pre-treated cells exhibited slightly larger/elongated morphology when compared to the untreated cells. After 6 weeks of osteogenic culture, cells in both groups were uniformly distributed throughout the scaffolds (Fig. 4b–b’’’), where cells were seen growing along with the internal lamellae pore structure. The MI192 pre-treated cells appeared to possess a slightly more elongated morphology compared to the untreated cells.

The influence of silk scaffold concentration on hDPSCs osteogenic differentiation was initially assessed by quantifying ALPSA following 2 weeks of osteogenic culture (Fig. 4c). Within the 2 wt% silk scaffolds, the MI192 pre-treated cells exhibited significantly elevated ALPSA when compared to the untreated cells (2.17 times) (*P* ≤ 0.001). A similar profile was observed in the 5 wt% group, where MI192 pre-treatment significantly increased ALPSA in hDPSCs (1.93 times) compared to the untreated control (*P* ≤ 0.001). The MI192 pre-treated and untreated cells within the 5 wt% scaffolds elicited a significantly enhanced ALPSA compared to the cells in the 2 wt% group (*P* ≤ 0.001). Therefore, the 5 wt% silk scaffolds were used for further analysis.

### MI192 upregulated hDPSCs osteoblast-related gene expression within 5 wt% silk scaffolds in vitro

**Fig. 5:**
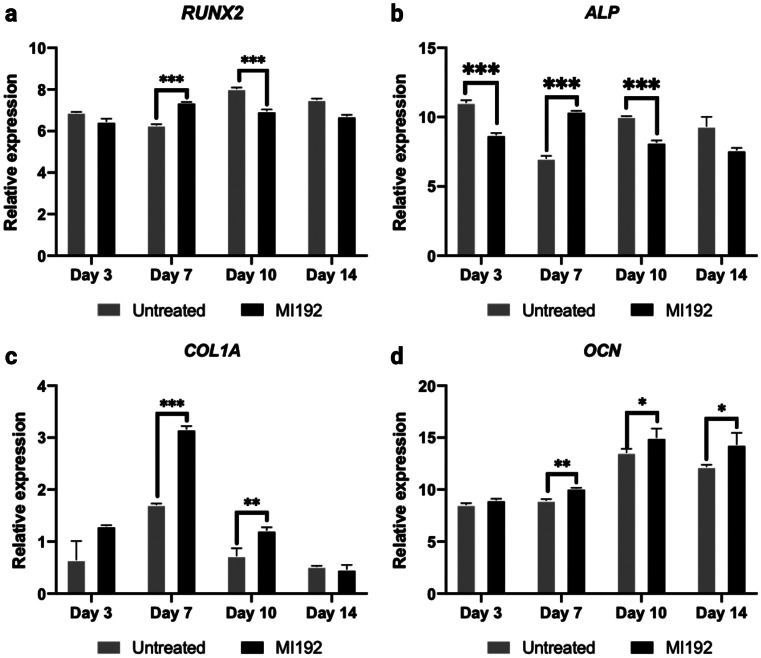
The effects of MI192 pre-treatment on the expression of osteoblast-related genes (**a**-**d**) in hDPSCs cultured in 5 wt% silk scaffolds during osteogenic culture. **a** *RUNX2*, **b** *ALP*, **c** *COL1A*, **d** *OCN*. Gene expression was relative to the housekeeping gene GAPDH. Data represented as mean ± SD (*n* = 3). *P ≤ 0.05, **P ≤ 0.01 and ***P ≤ 0.001

RT-qPCR showed the expression of the osteoblast-related genes (e.g. *RUNX2*, *ALP*, *COL1A*, *OCN*). The results showed that *RUNX2* mRNA expression was slightly reduced in the MI192 pre-treated cells compared to the untreated group on day 3 (*P* > 0.05) (Fig. 5a). After 7 days of culture, there was a significant upregulation in *RUNX2* expression in the MI192 pre-treated group compared to the untreated control (*P* ≤ 0.001). However, *RUNX2* expression in the MI192 group was significantly downregulated compared to the untreated cells on day 10 (*P* ≤ 0.001). *ALP* gene expression levels significantly decreased in the MI192 group when compared to the untreated cells after 3 days of osteogenic culture (*P* ≤ 0.001) (Fig. 5b). On day 7, the MI192-treated cells exhibited a significant increase in *ALP* expression than the untreated control (*P* ≤ 0.001). Following this, there was a significant reduction in *ALP* mRNA levels in the MI192 group on day 10 (*P* ≤ 0.001). In comparison, the MI192 pre-treated cells exhibited significantly enhanced *COL1A* gene expression on both days 7 (*P* ≤ 0.001) and 10 (*P* ≤ 0.01) compared to the untreated cells, whilst no significance was observed on days 3 and 14 (*P* > 0.05) (Fig. 5c). The *OCN* mRNA levels were similar on day 3 (*P* > 0.05). However, the MI192-treated group displayed significantly upregulated expression of OCN compared to the untreated control on days 7, 10 and 14 (*P* ≤ 0.01, *P* ≤ 0.05 and *P* ≤ 0.05, respectively) (Fig. 5d).

### MI192 enhanced hDPSCs osteogenic protein deposition and mineralisation within 5 wt% silk scaffolds in vitro

**Fig. 6:**
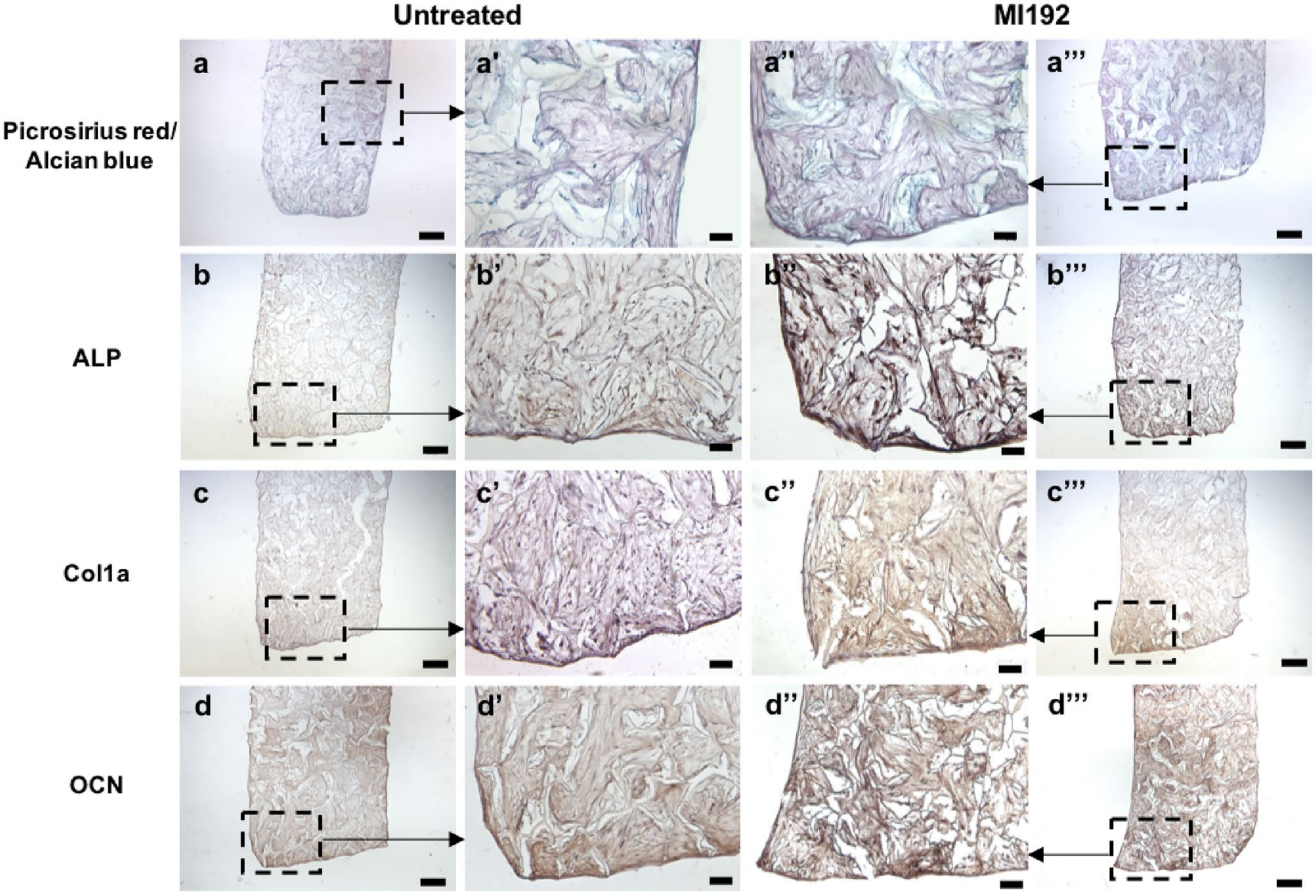
Histological staining for picrosirius red and alcian blue (**a**–**a’’’**) and immunohistochemical staining for ALP (**b**–**b’’’**), Col1a (**c**–**c’’’**) and OCN (**d**–**d’’’**) of MI192 pre-treated/untreated hDPSCs on 5 wt% silk scaffolds (*n* = 3) after 6 weeks osteogenic culture in vitro. Low magnification scale bars = 200 μm (**a**–**d** and **a’’’** to **d’’’**), and high magnification scale bars = 50 μm (**a’** to **d’** and **a’’** to **d’’**)

After 6 weeks in osteogenic culture, the histological assessment showed that the samples in both groups exhibited strong picrosirius red staining for collagen deposition throughout, with the strongest intensity situated at the periphery of the scaffolds (Fig. 6a–a’’’). The MI192 pre-treated group (Fig. 6a’’ and a’’’) displayed stronger global collagen staining throughout when compared to the untreated group (Fig. 6a and a’’’). Negligible staining for GAGs highlighted by alcian blue staining was observed in both constructs.

Immunohistochemical analysis showed that both MI192 pre-treated and untreated hDPSCs-silk constructs exhibited positive staining for the osteoblast-released markers assessed (ALP, Col1a and OCN), with increased protein depositions observed at the periphery of the scaffold in both groups (Fig. 6b–d). The MI192 pre-treated constructs showed substantially increased protein deposition throughout the scaffolds when compared to the untreated constructs.

**Fig. 7:**
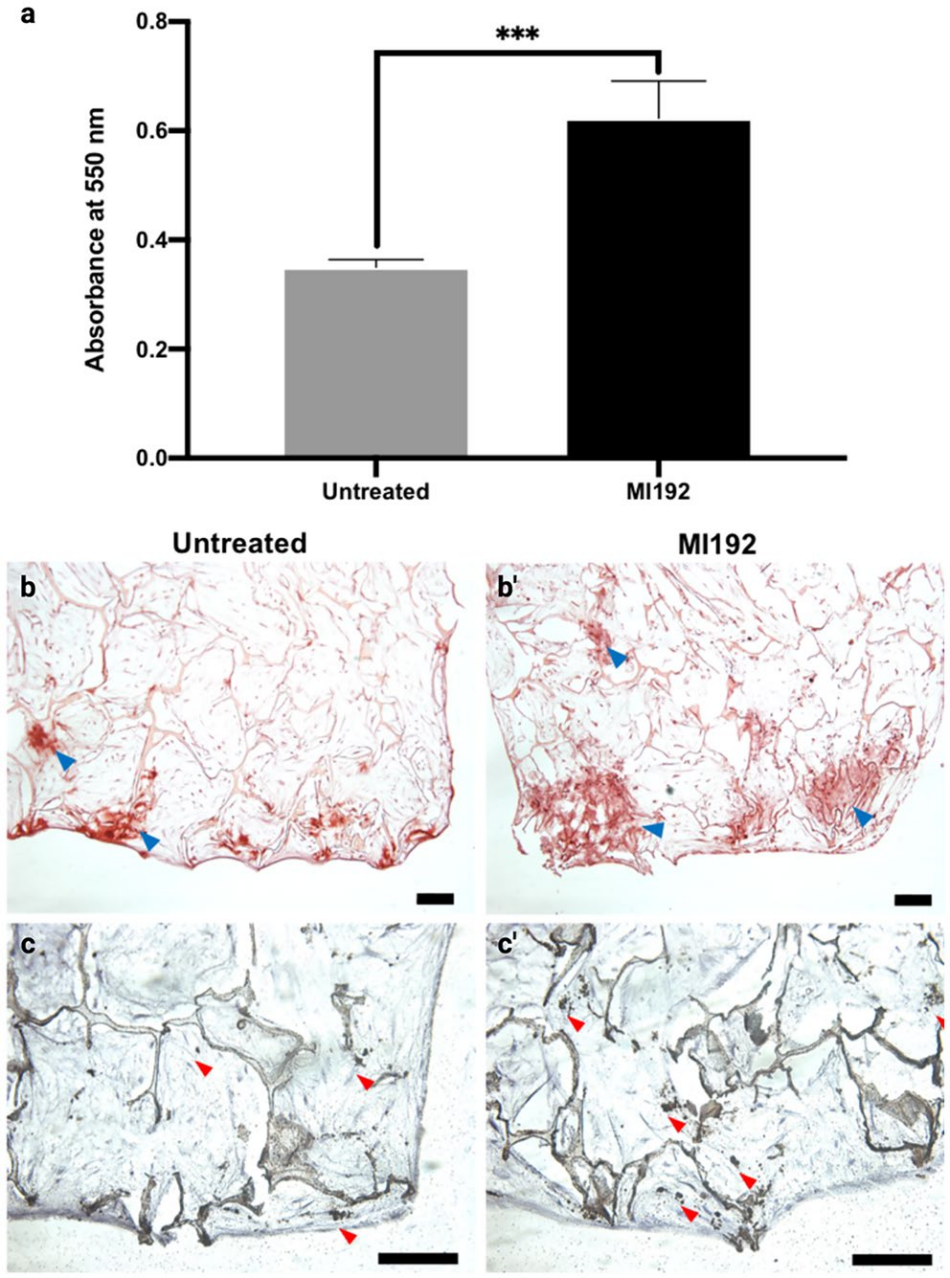
Effects of MI192 pre-treatment on hDPSCs calcium accumulation and mineralisation within 5 wt% silk scaffolds after 6 weeks osteogenic culture. **a** Quantitative alizarin red analysis, **b** & **b’** alizarin red staining (red colour: blue arrowheads), **c** and **c’**) Von Kossa staining (black colour: red arrowheads). Data represented as mean ± SD (*n* = 3). ***P ≤ 0.001. Scale bar, 100 μm

The effects of MI192 pre-treatment on hDPSCs calcium deposition within the silk scaffolds were determined by alizarin red staining. The MI192 pre-treated group exhibited a significantly increased quantity of calcium deposition (1.78 fold) compared to the untreated control (*P* ≤ 0.001) (Fig. 7a). The strongest accumulation of calcium deposits was situated towards the outer regions of both constructs (Fig. 7b and b’). To assess mineral nodule formation within the scaffolds, Von Kossa staining was conducted. Mineralisation was observed in both groups, particularly with increased density at the periphery of the scaffolds (Fig. 7c and c’). An enhanced quantity of black nodules (red arrows) were observed within the MI192 pre-treated constructs (Fig. 7c’) when compared to the untreated constructs (Fig. 7c).

### MI192 promoted hDPSCs extracellular bone matrix deposition and mineralisation within 5 wt% silk constructs in vivo

**Fig. 8:**
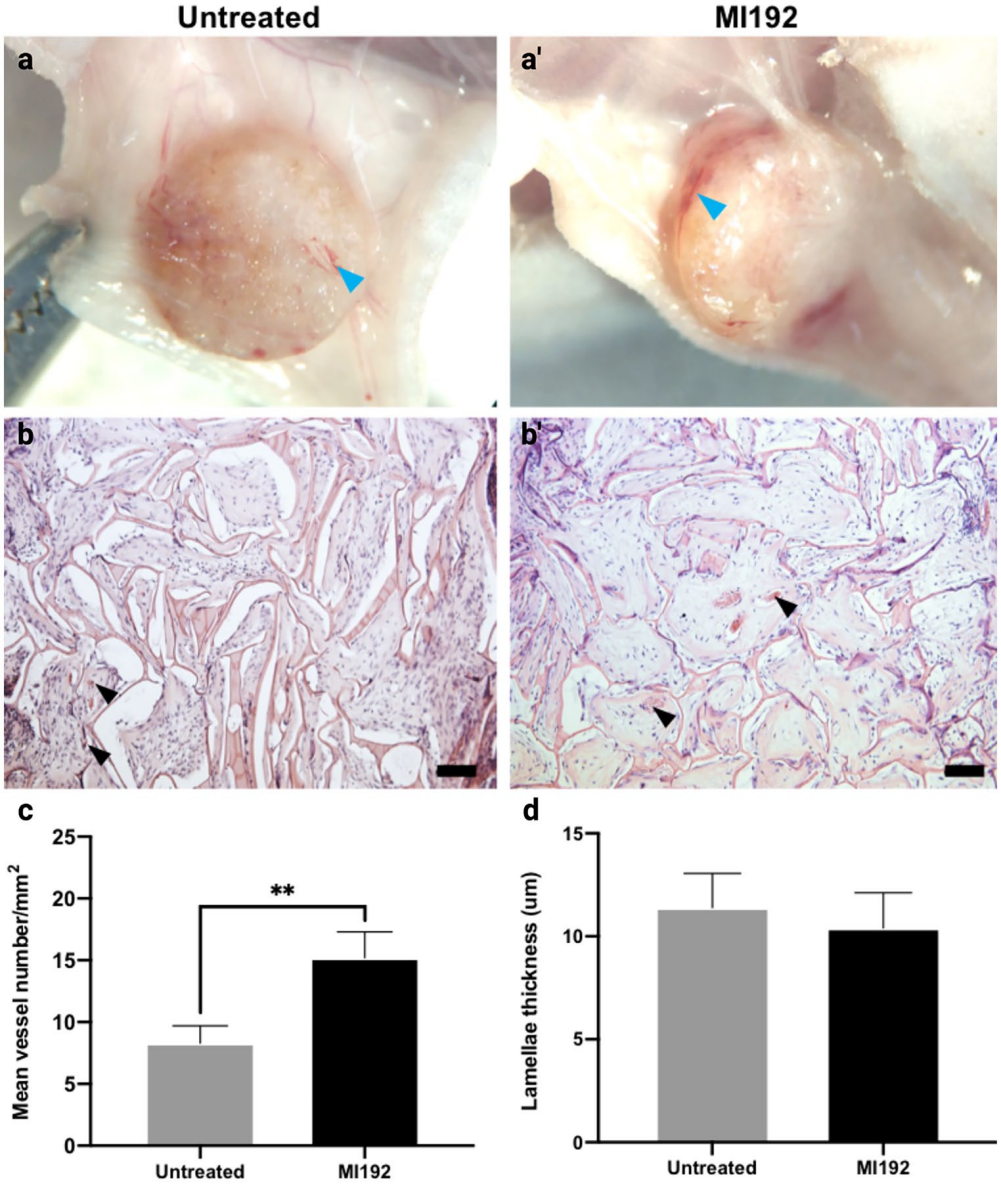
Macroscopic and histological analysis for angiogenesis and scaffold degradation of MI192 pre-treated hDPSCs-silk constructs following 6 weeks subcutaneous implantation in mice. **a** and **a’** Macroscopic image of cell-laden construct immediately prior to extraction. Light blue arrowheads highlight blood vessel formation. **b** and **b’** H&E staining showing hDPSCs throughout the silk constructs with subcutaneous tissues at the periphery. Black arrowheads highlight blood vessel infiltration. Scale bars = 100 μm. **c** Blood vessel density in hDPSC-silk scaffolds quantified from histological images. **d** Internal lamellae thickness. Data represented as mean ± SD (*n* = 3). ** *P* ≤ 0.01

The subcutaneous tissue surrounding the constructs was observed through macro imaging, with evidence of vascular infiltration into the scaffolds (blue arrow) (Fig. 8a and a’). H&E staining showed that cells were distributed homogenously throughout the scaffolds, with evidence of blood vessel infiltration into the scaffolds (black arrows) in both groups (Fig. 8b and b’). Quantification of these histological images indicated a significant increase in blood vessel density within the MI192 pre-treated constructs (1.82 fold) when compared to that in the untreated constructs (*P* ≤ 0.01) (Fig. 8c). There were 15.1 ± 2.1 vessels/mm^−2^ within the MI192-treated group, whilst the untreated group exhibited 8.3 ± 1.4 vessels/mm^−2^. There was a slight decrease in the scaffold lamellae thickness in the MI192 group when compared to that of the untreated group, although the decrease was not significant (Fig. 8d) (*P* > 0.05).

**Fig. 9:**
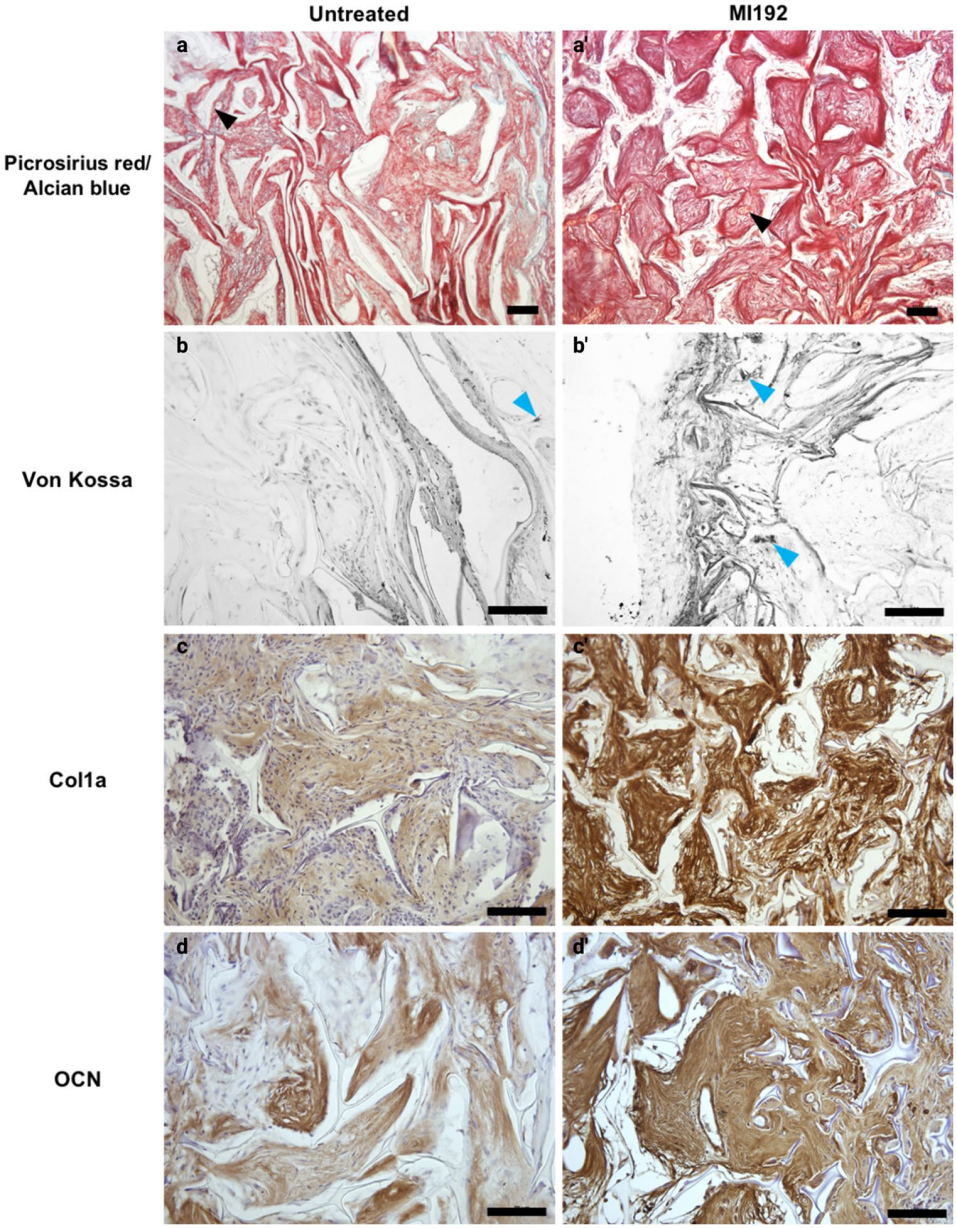
Histological and immunohistochemical staining of extracellular bone matrix and mineralisation of MI192 pre-treated hDPSCs within silk constructs following 6 weeks subcutaneous implantation in mice. Positive histological staining for collagen (red colour, picrosirius red/alcian blue staining) and black arrowheads indicated vessel formation (**a**, **a’**). Mineral nodule formation (black colour — light blue arrows, Von Kossa staining) (**b**, **b’**). Production of type I collagen (**c**, **c’**) and OCN (**d**, **d’**) proteins was evaluated via immunohistochemical staining (brown colour). Scale bar, 100 μm

The effects of MI192 pre-treatment on hDPSCs extracellular matrix deposition and mineralisation within constructs following subcutaneous implantation was investigated via histological analysis (Fig. 9). Picrosirius red/alcian blue staining showed that both groups possessed strong staining for collagen deposition, with increased intensity observed in the MI192 group (Fig. 9a and a’). Von Kossa staining confirmed that MI192 pre-treatment enhanced nodule formation compared to the untreated constructs (Fig. 9b and b’). Immunohistochemical analysis showed the effects of MI192 on hDPSCs extracellular matrix production within silk scaffolds in vivo. Positive immunostainings for Col1a (Fig. 9c and c’) and OCN (Fig. 9d and d’) were observed in both MI192 pre-treated and untreated groups, with the MI192 pre-treated group exhibiting increased protein deposition compared to the untreated control.

## Discussion

Epigenetic modifications are known to control transcriptional activity, with HDACis demonstrating their potential for enhancing MSCs’ osteogenic capacity (Cho et al. 2005; Man et al. 2021b; Paino et al. 2014). The majority of studies have focussed on assessing the efficacy of these epigenetic modifying compounds in 2D in vitro culture, with limited use of 3D in vitro models. Over the last decade, there have been an increased number of reports on the use of HDACis for bone regeneration in vivo. For example, Lee et al. (2011) utilised a collagen scaffold and a calcium phosphate scaffold soaked with Largazole for the repair of a calvarial bone defect model (Lee et al. 2011). Similarly, Jung et al. (2010) combined a calcium sulphate scaffold with TSA to stimulate osteogenic differentiation in vitro and in vivo (Jung et al. 2010). Importantly, these studies have employed non-selective panHDACis, which exhibit reduced differentiation efficacy compared to selective HDACis and are associated with potential side effects from inhibiting a broad range of HDAC isoforms (Lawlor 2016; Yang et al. 2017). Hence, there is great precedence to shift towards the use of selective HDACis for bone augmentation strategies (Yamauchi et al. 2020). In this study, the efficacy of a selective HDAC2 and 3 inhibitor — MI192 to stimulate hDPSCs osteogenic capacity within the *Bombyx Mori* lyophilised silk scaffolds — was investigated in vitro and in vivo.

Due to silk’s favourable properties for tissue engineering (Melke et al. 2016; Sun et al. 2021), it has been increasingly combined with epigenetic strategies for bone regeneration. For example, Han et al. (2015) reported hBMSCs overexpressing the H4K20/H3K9 demethylase PHF8, accelerated craniofacial and bone development when delivered within a silk fibroin scaffold in mice (Han et al. 2015). Hence, the potential use of silk scaffolds to facilitate epigenetically enhanced MSCs for bone repair has been demonstrated. In this study, we have found that the silk scaffolds displayed a highly porous surface topography, which is similar to the data reported in the literature (Saha et al. 2013; Uebersax et al. 2006; Varkey et al. 2015). Moreover, these scaffolds exhibited a thin sheet-like lamellae network, where increasing the silk concentration resulted in thicker lamellae. This is expected as more ice crystals form within the negative cast of the pores during the fabrication process, thus displacing the silk dissolved in the solution and creating a concentrated silk layer surrounding the fully formed crystal (Joukhdar et al. 2021). Hence, the increased lamellae thickness of the 5 wt% scaffold can be attributed to an increased quantity of silk available to concentrate around the ice crystals during fabrication. Mechanical characterisation showed that the 5 wt% silk scaffold was stronger and less elastic with compression modulus approximately fourfold higher than the 2 wt% scaffolds, consistent with previous findings (Rnjak-Kovacina et al. 2015).

Additionally, in vitro degradation of such silk scaffolds was a function of the silk concentration, where the 2 wt% scaffolds degraded at a much higher rate compared to the 5 wt% group following exposure to protease XIV, correlating with the findings in the literature (Rnjak-Kovacina et al. 2015). The enhanced mechanical properties exhibited by the higher percentage scaffold is beneficial for bone tissue engineering applications in not only supporting the defect site immediately post-implantation but also promoting MSCs osteogenic differentiation through increased mechanotransductive stimulation (Ghasemi-Mobarakeh et al. 2015; Sun et al. 2018). Moreover, numerous studies have reported the role of biomaterials in augmenting important epigenetic functions via the modification of the cell’s actin cytoskeleton that transmits forces to the nuclear protein lamin A/C, ultimately resulting in increased transcriptional activation (Downing et al. 2013; Li et al. 2011). For example, 3D-printed titanium scaffolds exhibiting a triangle pore shape significantly enhanced osteoblast mineralisation via hyperacetylation-induced gene activation when compared to cells on 3D-printed constructs exhibiting a square pore conformation (Man et al. 2021d). Altogether, these findings indicate the importance of silk concentration in modulating the key physical properties of the scaffolds, ultimately impacting its potential clinical utility for bone augmentation strategies.

The behaviour of MSCs is tightly regulated by the microenvironments in which they reside, where the external factors affect the key cellular processes such as proliferation and differentiation (Breuls et al. 2008; Maioli et al. 2018). ALP activity, an early marker of osteogenesis, was assessed to initially determine the influence of the silk scaffold concentration on hDPSCs osteogenesis with/without MI192 pre-treatment. In both scaffold types, MI192 pre-treated hDPSCs exhibited a significantly elevated ALPSA compared to the untreated cells, consistent with the treatment with MI192-enhanced hDPSCs ALPSA observed in 2D in vitro (Man et al. 2021b). Interestingly, the MI192-induced increase in hDPSCs ALPSA was slightly more remarkable in the silk scaffolds (~ 2 folds) when compared to that in 2D culture (1.68 fold), likely due to the influence of the scaffold 3D microenvironment on promoting osteogenesis (Ghasemi-Mobarakeh et al. 2015; Sun et al. 2018). These findings demonstrate that MI192 is capable of promoting hDPSCs osteogenic differentiation within this 3D culture model, correlating with the enhanced ALPSA of MI192-treated hBMSCs when cultured as bio-assembled microtissues (BMT) (Man et al. 2021c). Moreover, our findings also showed that ALPSA of both MI192 pre-treated and untreated cells were significantly increased in the 5 wt% silk scaffolds compared to their respective cells in the 2 wt% silk scaffolds (1.27 and 1.44 fold, respectively). These results imply that the increased stiffness exerted by increasing the polymer concentration plays an important role in modulating the osteogenic capacity of hDPSCs (Engler et al. 2006; Witkowska-Zimny et al. 2013). These findings also indicate the importance of silk concentration not only for supporting the bone defect site mechanics but also in stimulating MSCs differentiation (Ghasemi-Mobarakeh et al. 2015; Sun et al. 2018). Therefore, the 5 wt% construct was utilised to further assess the effects of MI192 on promoting hDPSCs osteogenic differentiation within the silk scaffolds.

Several studies have reported enhanced transcriptional activity and gene activation induced by HDACis (Jin et al. 2013; Schroeder et al. 2004). Although the efficacy of MI192 to stimulate hDPSCs osteoblast-related gene expression has been shown in 2D culture (Man et al. 2021b), it is important to evaluate its ability to promote osteogenesis within a more physiologically relevant environment. Our findings showed that MI192 accelerated the expression profiles of the early osteogenic genes *Runx2* and *ALP* within hDPSCs compared to the untreated control. Moreover, the expressions of the mid-late stage osteogenic genes *Col1a* and *OCN* were also significantly upregulated in MI192 pre-treated cells within the silk scaffolds during osteoinductive culture. These findings show the capability of MI192 in enhancing the expression of osteoblast-related markers, which are integral in promoting hDPSCs lineage-specific differentiation (Jonason et al. 2009; Sasano et al. 1993). The increased osteoblast-related gene expression observed in this study is consistent with the findings from MI192 pre-treated hDPSCs in 2D culture (Man et al. 2021a) and MI192 pre-treated hBMSCs in the BMT model (Man et al. 2021c). Collectively, these results suggest the plasticity of MI192 in promoting the osteogenesis of MSCs derived from different tissues and cultured in various model systems. It is important to note that MI192-enhanced hDPSCs osteogenesis is likely due to the increased chromatin’s transcriptional permissiveness similar to panHDACis mechanism. However, due to its selective inhibition of HDAC3, such effects may result in enhancing the transcriptional activity and stability of Runx2 (Jonason et al. 2009). Therefore, it is probable that the MI192 pre-treated cells exhibited increased transcriptionally active Runx2, although further investigation is required.

Histological analysis was conducted to evaluate the effects of MI192 on stimulating hDPSCs extracellular matrix deposition within the silk scaffolds. The MI192 pre-treated group exhibited substantially increased collagen deposition confirmed via picrosirius red staining when compared to the untreated control, similar to the work performed previously with MI192 pre-treated ADSCs on *Antheraea mylitta* (AM) silk scaffolds (Lawlor 2016). Further evaluation by immunohistochemical analysis showed that both untreated and MI192 pre-treated constructs exhibited positive expression of osteoblast-related proteins ALP, Col1a and OCN. Interestingly, the MI192 pre-treated constructs displayed substantially increased expression for all these assessed osteogenic markers compared to the untreated group, consistent with the upregulated osteogenic gene expression and ALPSA findings in this study. Similarly, it was also reported that MI192 promoted the osteoblast-related protein expression of hBMSCs when cultured in the BMT model (Man et al. 2021c). Paino et al. (2014) demonstrated that valproic acid (VPA) pre-treatment promoted hDPSCs collagen deposition whilst inhibiting OCN protein expression in lyophilised collagen type I scaffolds (Paino et al. 2014), indicating the effect of VPA in inhibiting terminal differentiation of hDPSCs. Intriguingly, this was not observed in our study, likely due to the increased HDAC isoform selectivity of MI192 towards HDAC3 compared to VPA. Previously, it was shown that MI192 enhanced ADSCs expression of Col1a but not OCN in Antheraea mylitta (AM) silk scaffolds (Lawlor 2016), which differed from the findings in this study. Probably, the higher wt% scaffolds and the different MSCs type utilised in this study resulted in the elevated expression of OCN. The enhanced deposition of osteoblast-related extracellular matrix proteins following osteogenic culture is likely facilitated by the slow-on/off binding kinetics of MI192 to HDAC isoforms when compared to other HDACis as reported by Boissinot et al. (Boissinot et al. 2012). Therefore, the slow-binding kinetics of MI192 possibly potentiates the augmented epigenetic function within the pre-treated hDPSCs in silk scaffolds during osteogenic culture, similar to the findings observed in 2D culture (Man et al. 2021b). Thus, these findings demonstrate that MI192 pre-treatment is able to stimulate hDPSCs deposition of osteoblast-related extracellular matrix proteins within silk scaffolds in vitro.

The effects of MI192 on hDPSCs calcium deposition and mineralisation were assessed via alizarin red and Von Kossa staining, respectively. Both groups displayed positive calcium deposition within the scaffolds. However, the MI192 pre-treated cells exhibited significantly increased calcium content (1.78 fold) compared to untreated cells. A similar profile was observed following Von Kossa staining, and the MI192 pre-treated group displayed substantially enhanced mineral nodule formation. These findings correlate well with the enhanced mineralisation observed with the MI192 pre-treated ADSCs in the AM silk scaffolds (Lawlor 2016) and VPA pre-treated hDPSCs in the Gingistat scaffolds (Paino et al. 2014). The areas with the most significant calcium and mineral deposition in the scaffolds corresponded to the regions of increased extracellular protein expression, indicating cells of a more mature osteogenic phenotype in these locations. A number of studies showed that the extracellular matrix proteins sequester bioactive factors in the cells’ secretome involved in mineral nucleation (Blaser and Aikawa 2018; Man et al. 2020). Interestingly, extracellular vesicles (EVs) derived from epigenetically-modified osteoblasts were shown to significantly increase MSCs matrix mineralisation when compared to EVs from unmodified cells (Man et al. 2021a). Hence, it is likely the secretome plays a critical role in promoting extracellular matrix mineralisation of MI192 pre-treated hDPSCs within the scaffolds.

There have been limited reports investigating the efficacy of HDACis to promote MSCs osteogenic differentiation in vivo (Huynh et al. 2017; Jung et al. 2010; Lee et al. 2011), and the majority of these studies have harnessed panHDACis, which are associated with several issues (Lawlor 2016; Yang et al. 2017). In the present study, the effects of MI192 on stimulating hDPSCs bone-like tissue formation within the silk scaffolds were assessed in vivo via subcutaneous implantation. Vascularisation of scaffolds targeted for critical-sized defects is an essential requirement for developing functional bone constructs, as the lack of necessary nutrient/waste exchange may lead to necrosis within the scaffold and ultimately result in inadequate integration into the host tissues (Rouwkema et al. 2008). Vascular ingrowth from the surrounding subcutaneous tissues was observed via histological analysis in both groups, indicating the silk scaffold provides a favourable microenvironment for vascularisation, which has been similarly shown in the literature (Han et al. 2016; Lin et al. 2020). Interestingly, we reported a significant increase in the blood vessel density within the MI192 pre-treated cells in the silk scaffolds compared to the untreated cells. This may indicate the role of HDACi treatment on augmenting the secreted trophic factors, such as cytokines or EVs from the hDPSCs in vivo, resulting in enhanced recruitment of endothelial cells into the scaffolds (Diomede et al. 2018; Man et al. 2020), although this would require further investigation. Park and co-workers (2010) reported that the silk scaffold degradation rate is linked to improved bone-like tissue formation (Park et al. 2010). Histological analysis showed a reduced scaffold volume in the MI192 pre-treated constructs compared to the untreated control, indicating enhanced de novo tissue formation within the scaffolds.

Moreover, faster scaffold degradation rates have been suggested to facilitate vascular infiltration and vessel formation (Zhang et al. 2019), consistent with our vascularisation findings. The MI192 pre-treated groups exhibited increased extracellular matrix deposition observed through picrosirius red collagen staining and immunostaining of Col1a and OCN, similar to our in vitro data, providing further evidence supporting the efficacy of this HDACi in promoting hDPSCs osteogenic capacity. Importantly, the MI192 pre-treated constructs exhibited an increased quantity of mineral nodules. The enhanced extracellular matrix deposition and mineral formation observed from the MI192 pre-treated hDPSCs were consistent with the elevated collagen production and mineral deposition observed from MI192 pre-treated ADSCs within AM silk scaffolds in vitro (Lawlor 2016). Similar results were found in the MI192 pre-treated hBMSCs in the BMT model in vitro and in vivo (Man et al. 2021c). These observations indicate that MI192 is capable of promoting the osteogenic lineage-specific differentiation of MSCs derived from different stem sources and cultured in different scaffold systems in vitro and in vivo. Taken together, these findings demonstrate lyophilised silk scaffolds provide a suitable platform to facilitate the delivery of MI192 pre-treated hDPSCs in vivo, enhancing its efficacy to stimulate de novo bone formation.

Looking forward, it is necessary to investigate the capacity of MI192 pre-treated hDPSCs within silk scaffolds to stimulate critical-sized bone defect repair, which would provide greater pre-clinical evidence into the translation of this cell-based approach for effective functional bone regeneration in the clinical setting.

## Conclusion

In conclusion, this study provides evidence that the selective HDAC2 and 3 inhibitor MI192 is capable of promoting hDPSCs osteogenic differentiation within lyophilised *Bombyx Mori* silk scaffolds in vitro and in vivo, indicating the potential of combining epigenetic reprogramming with porous silk scaffold for enhancing the efficacy of MSCs-based therapies for bone augmentation strategies.

## Supplementary Information

Below is the link to the electronic supplementary material.

Supplementary file1 (DOCX 14 KB)
