# Supplementary material for: Bone tissue engineering using 3D silk scaffolds and human dental pulp stromal cells epigenetic reprogrammed with the selective histone deacetylase inhibitor MI192
Source: Cell Tissue Res. 2022 Apr 1;388(3):565–81. doi: 10.1007/s00441-022-03613-0 (PMC9110470; doi:10.1007/s00441-022-03613-0)
Supplement: Supplementary file 1 — Supplementary file1 (DOCX 14 KB) [file 441_2022_3613_MOESM1_ESM.docx]

**Supplementary materials**

**Supplementary Table 1 - Primers utilised for Taqman RT-qPCR**

| **Gene symbol** | **Description** | **TaqMan**  **gene primer** |
| --- | --- | --- |
| ***GAPDH*** | Glyceraldehyde-3-phosphate dehydrogenase | Hs99999905_m1 |
| ***RUNX2*** | Runt-related transcription factor 2 | Hs00231692_m1 |
| ***ALPL*** | Alkaline phosphatase | Hs01029144_m1 |
| ***COL1A1*** | Collagen type I, alpha 1 | Hs00164004_m1 |
| ***OCN/BGLAP*** | Osteocalcin/ PMF-bone gamma- carboxyglutamate (gla) protein | Hs00609452_g1 |

**Supplementary Table 2 - Antibodies used for immunohistochemical staining**

| **Antibody** | **Catalogue number** | **Concentration** |
| --- | --- | --- |
| ***In vitro*** |  |  |
| **Alkaline Phosphatase**  (Abcam) | ab126820 | 1/500 |
| **Collagen type I**  (Abcam) | ab6308 | 1/100 |
| **Osteocalcin**  (Abcam) | ab13420 | 1/800 |
| ***In vivo*** |  |  |
| **Collagen type I**  (Abcam) | ab138492 | 1/100 |
| **Osteocalcin**  (Abcam) | ab198228 | 1/200 |
